# Ayurveda–modern medicine interface: A critical appraisal of studies of Ayurvedic medicines to treat osteoarthritis and rheumatoid arthritis

**DOI:** 10.4103/0975-9476.72620

**Published:** 2010

**Authors:** Arvind Chopra, Manjit Saluja, Girish Tillu

**Affiliations:** *Center for Rheumatic Diseases, Pune, India*

**Keywords:** Ayurveda, rheumatoid arthritis, osteoarthritis, clinical trials, *Rasayana*

## Abstract

The potential of Ayurvedic philosophy and medicines needs to be recognized and converted into real life treatment paradigm. This article describes a comprehensive therapeutic approach used in Ayurveda and modern medicine to treat arthritis. We present concise summary of various controlled drug trials carried out by us to validate standardized Ayurvedic drugs using modern medicine protocol to treat Rheumatoid Arthritis and Osteoarthritis knees. Several of the latter are published. The trials consistently demonstrate excellent safety of Ayurvedic medicines but often fail to unequivocally show superior efficacy. Some key findings of a recently unpublished trial in OA knees are also presented to show equivalence between Ayurvedic medicine and celecoxib and glucosamine, and we speculate that equivalence trials may be a way forward. The data from the trials also supports the Ayurvedic *‘Rasayana’* concept of immune-modulation and healing. We need to interpret logic of Ayurveda when, adopting modern science tools in drug development and validation and much research is required. Validation of Ayurvedic medicines using the latter approach may lead to an evidence based Ayurveda – Modern Medicine interface. Also, in pursuit of finding better treatment solutions, we ought to step beyond the realm of only drugs and attempt validation of comprehensive specific treatment package as per classical Ayurveda. Finally, validation of a combined (Ayurveda and modern medicine) therapeutic approach with superior efficacy and safety is likely to be a major leap in overcoming some of the current frustrations to treat difficult disorders like arthritis using only modern medicines.

## INTRODUCTION

Ayurveda is an indigenous ethnic medical system in popular practice in the Indian subcontinent since the pre-biblical era.[1–4] The system’s core strength is its holistic approach to health and disease using natural remedies derived from medicinal plants and minerals. Laying emphasis on self-discipline and modest living with high human values, the system strongly advocates a unique set of principles and guidelines on diet and exercise in daily healthy living.

The classical Ayurvedic system was probably driven by insight, intuition, and astute observation of human behavior and nature. “The soil is more important than the seed” concept underlies several Ayurvedic treatment strategies. A delicate balance between biophysiological forces (*dosha*) and constitution (*prakriti*) is said to determine health and disease; several other “players” like “mind” and “metabolic fire” (*agni*) play important roles. Ayurveda’s principle therapeutic aim is to harmoniously restore that balance. Man is regarded as bearing a structure transcending all matter in the universe, animate and inanimate. This commonality makes “man a replica of the universe.” Plants are no exception, all matter has medicinal value.

Ayurveda has an extensive pharmacopoeia, predominantly herbs and minerals. Their healing properties are well summarized in modern texts.[5] Ayurvedic formulations, often complex with several herbal-mineral ingredients, are governed by well-described pharmacological principles of preparation, compatibility and administration. In some complex, well-controlled physicochemical processes convert raw metals and minerals into potent medicines known as *bhasmas*. Although classic texts contain descriptions of classic formulations, traditional Ayurvedic practitioners often modify them to suit the individual constitution (*prakriti*), which confers genetic predisposition toward disease and therapy response, and is vital to ensure medication safety. Safety is inherently bound to efficacy, forming an important endpoint when preparing an Ayurvedic formulation. A formulation’s medicinal power is a function of its milieu interior, not merely due to any single plant extract. Molecular structure as viewed in modern science is not described, and is of no particular significance in determining a formulation’s therapeutic properties. Ayurveda’s basic perspective: “no two individuals are alike” holds, even when they suffer from seemingly similar ailments. Also, advice on diet, exercise, and lifestyle are inherently bound to its basic therapeutic approach. Rather than seeking support from laboratory or imaging investigations, Ayurvedic physicians use subtle clinical methods to diagnose and monitor therapeutic response.

Biomedicine, in contrast, is founded on the reductionist approach to health and disease, and attempts, first and foremost, to eliminate pathology. Although clinical evaluation is of paramount and critical importance, science as such is extremely impersonal, and, when treating patients, generally cuts across individual differences (genetic or other). Proneness to disease and prevention thereof are more environmental and genetic issues than questions of “wholesome strengthening of the host.” There is a strong, across-the-board underpinning of “objectivity,” in diagnosis, treatment, and therapy response. Medicines are its core strength – well-characterized in structure and function (usually well-tested under laboratory and clinical trial conditions), with efficacy/safety tradeoffs. Response is generally predictable.

Believing that common ground between the two systems should be explored using modern science and logic to understand Ayurveda’s ancient thought and system processes, we offer this critical appraisal using “arthritis” as a model disorder.[6,7] We first review Ayurveda’s perspective on “arthritis”; next, based on our experience of Ayurvedic drug trials carried out since 1996, we discuss issues critical to developing and validating an Ayurveda–biomedicine interface; finally, we conclude with some futuristic thoughts and ideas.

## ARTHRITIS – AN AYURVEDA DISCRIPTION

Undoubtedly, precise translation of Ayurvedic nomenclature into modern medical terminology is difficult. However, distinctions are made between different articular disorders, descriptions of which bear resemblance to Rheumatoid Arthritis (RA) and Osteoarthritis (OA). In many Indian languages, Vata, distinct from *vata dosha*, is a common colloquial term used to denote rheumatism. When primarily affecting joints, it is often called “*sandhivata” (sandhi*=joint). Many forms of arthritis were described along with the nervous system disorders in the classic texts.[8,9] The condition of Amavata[10] has been described as a dreadful, painful, swollen polyarticular affection similar to RA. *Vata dosha* plays a major role in the causation of arthritis. Joints and soft tissues are affected by “*ama*”, produced in the gut due to “weakened” agni, food indiscretions, or disturbed *dosha* equilibrium, resulting in inflammatory and obstructive processes. In Ayurveda, “arthritis is linked to the gut”: Ayurvedic formulations invariably target joints, gut, *and* immune systems. How intriguing, even surprising, that thousands of years later, modern medicine should find such an essential immune-mediated link between certain gut disorders and inflammatory arthritis!

Several publications support purported anti-inflammatory and biologic effects of some popular anti-arthritic Ayurveda medicinal plants,[11–16] demonstrating immunomodulation. Such an immunologic basis is conceptually captured by the “*Rasayana*” (means “strengthening and rejuvenating”) branch of Ayurvedic science.[4,6] Ayurvedic pharmacopeias[10] contain lucid descriptions of *Rasayana* properties of medicinal herbs and minerals, several of which are used to treat arthritis.

*Rasayana* aims to increase the body’s resistance to disease (*vyadhi-kshamatva*), delay aging, and promote body strength and intellect. *Rasayana* practices in daily life are rejuvenating and in disease promote healthy recovery. The prime example of a *Rasayana* plant is *Withania somnifera (Aswagandha)*,[17–20] extensively used in Ayurvedic medicine, and often compared to Ginseng; its immunomodulatory, anti-inflammatory, and hence anti-arthritic, and other biologic effects have been extensively documented.

*Ricinus communis (Erand*/castor oil) and *Guggul* extracts (*Commiphora mukul, Boswellia serrata*) are prime examples of potent anti-arthritic medicinal plants named in Charaka Samhita (CS). Numerous other *Rasayana* plants, especially *Withania somnifera*, are common components of anti-rheumatic medications. Other well-standardized formulations manufactured on a large scale by the Indian pharmaceutical industry are *Dashamool, Mahayograj Guggul, Vatavidhwansa, Suvarna Bhasma, Guggul, Yograj Guggul*, and *Triphala Churana*. Several of these have potent laxative action. *Guggul* preparations often contain ash (*Bhasma*) of minerals such as gold (*Suvarna Bhasma*), silver, copper, iron, mica, mercury, sulfur, zinc, lead.

It is fascinating that “gold” in its Ayurvedic ash form has been used to treat arthritis since ancient times, while modern medicine inadvertently discovered its use as disease-modifying anti-rheumatic drug (DMARD) in the last century. CS describes complex poultice preparations made by mixing herbs, minerals, and animal meat. Certain medicated massage oils like Bala taila,[21] also used in the treatment of arthritis, may contain more than 50 ingredients.

Treatment of arthritis usually begins with two basic processes: *snehana* (oleation) and *swedana* (sweating, heating). While diaphoretic, steam bath, may be used to carry out the latter, oily preparations are administered orally, through medicated enemas (Basti), or massage for oleation. These aim to cleanse and purify the body to restore *tridosha* equilibrium. Such drugs are administered to patients through multiple routes concurrently or sequentially. *Panchakarma (Five Processes)* comprises treatments curative to dosha imbalance, including emetics (*Vamana*), purgatives (*Virechana*), medicated oily enema (*Anuvasana Basti*), medicated decoction/dry enema (*Asthapana Basti/Niruhana*), and oleation/nasal purgation (*Shirovirechana/Nasya*). Guided by therapeutic response, *Panchakarma* procedures are indicated for specific stage of disease. They are widely used to treat many forms of arthritic conditions, including RA.

Dietary restrictions form the mainstay of treatment, and physical exercises and yoga are advocated at appropriate stages of recovery. Some patients are made to fast (*Langhana*) in initial, acute stages: the digestive and metabolic systems are strengthened and digest accumulated *Ama*. Similarly, patients are recommended special, easily digestible diets with attributes opposite to the offending *Dosha*. Application of leaches and venesection, recommended to remove excess *Dosha*, and relieve pain and swelling.[22]

Pain relief for painful, swollen joints is produced by local application of plant extracts (for example, *Semecarpus anacardium* or marking nut), which produce chemical cauterization and superficial burn-like reactions. Similar cauterization may be achieved by applying heated probes of gold, copper, or iron known as *Agnikarma*.

Ayurvedic massage is very popular and is said to have several effects – alleviating *vata*, removing subcutaneous fat, reducing fatigue and pain, and stimulating the nervous system. In Ayurvedic massage, selection of oils and technique is guided by the patient’s *Dosha* and *Dahtu status*. Ayurvedic pressure points (*marmas*), as in acupuncture, are well described, and used during massage to stimulate internal organs. Exercise is recommended to make the body light and flexible, and to enable it to withstand heat, cold, hunger, thirst, and fatigue.

*Yoga* has been described as the best means to achieve physical and mental fitness. *Yoga* and Ayurveda share common fundamental principles of anatomy, physiology, pathogenesis (including *tridosha*), and treatment, and complement each other. Traditional practitioners advocate use of both to treat chronic ailments, including arthritis.

Ayurveda aims to “cure” (*Aturasya-vyadhi-parimokshah*). Using *Rasayana*, the host must be strengthened to prevent relapses. The texts describe prognostications and limitations of therapy. For example, rheumatism is likely to be incurable if all three *doshas* are vitiated, and likely to persist for long time if two *doshas* are vitiated[23] In our experience, consensus among Ayurvedic physicians on how best to treat a particular form of arthritis is difficult to obtain. Differences are often attributable to patients’ diagnosis. Several components of Ayurvedic treatment of RA and OA may be similar.

## CLINICAL DRUG TRIALS

Drug trials described here[24–29] were carried out in accordance with protocols conforming to standard scientific requirements of modern medicine[30] Ayurvedic physicians were involved in each trial from its inception; eligible patients were screened and diagnosed according to standard rheumatology criteria; after enrollment their response was monitored by biomedical physicians and/or rheumatologists. Several Ayurvedic parameters including *prakriti* were also recorded in the more recent trials. Appropriate institution ethic board clearance and informed patient consent were obtained.

Ayurvedic formulations were usually selected through consensus of experts. Component plant extracts are well-described in Ayurvedic literature, and further characterized in Ayurvedic pharmacopeias. Formulations were properly standardized at all levels of manufacture, ranging from plant selection and procurement to HPLC chemical markers – mostly bioactive ones considered important mediators of therapeutic potential. Modern methods were used to prepare Ayurvedic tablets/capsules. Whenever required, starch containing placebo was carefully matched. GCP guidelines and other regulatory requirements were adhered to. All trials presented here were principally carried out, coordinated, and analyzed by CRD. Some recent trials were carried out under the “New Millennium Indian Technology Leadership Initiative” (NMITLI) arthritis project. There, clinical strategy was to move step-wise from simple exploratory evaluations to better-powered statistically designed drug trials of longer duration.

## RHEUMATOID ARTHRITIS: BIOMEDICAL DESCRIPTION

Rheumatoid arthritis[31] (RA) is the prototype of a severely painful chronic disease that affects multiple joints, causing swelling and crippling deformities in most patients. The world wide[32] prevalence is about 1%; recently we reported 0.3–0.7% in Indian population studies.[33] RA causes extra-articular systemic complications; it is a risk factor for premature atherosclerosis and coronary artery disease. It is predominantly seen in women belonging to the peri-menopausal age group and often leads to poor quality of life. Immediate pain relief is provided by analgesics (paracetamol/tramadol) and non-steroidal anti-inflammatory drugs (NSAIDs) like ibuprofen, diclofenac, and celecoxib. NSAIDs also alleviate joint swelling, but can cause deleterious effects in the gastrointestinal (esp. ulcer), renal, and cardiovascular systems. Oral steroids are potent anti-inflammatory agents, widely used in RA, but requiring great caution and discretion. Steroids notoriously cause a wide spectrum of toxicity that can even occur at relatively low doses over short periods of administration.

DMARD, such as the currently popular methotrexate, sulfasalazine, leflunomide, and chloroquin, are pivotal in controlling the disease process, its activity, and progression. Intended to induce remission, they may prevent or slow deformities. Their optimum effect, reached slowly over 2–3 months, reduces or even nullifies requirements for analgesics, NSAID, or steroids. DMARD can produce severe systemic toxicity, however, including infections (being immunosuppressive), and need careful clinical and laboratory monitoring. For better efficacy, they are often combined, not necessarily meaning more toxicity. Long-term patient compliance is poor; only 50–55% achieve meaningful disease control.

Powerful “biological DMARD agents” are now popular: essentially antibodies against cytokines (inflammatory mediators, TNF, a prime target) or immune cell surface receptors, they provide rapid control of RA. Infections (especially tuberculosis), high expense, and long-term compliance are chief concerns, however. Overall, 25–30% patients do not respond adequately, necessitating changes in biologic DMARD.

For modern medicine, RA management continues to present formidable challenges. The disease is lifelong; no cure is in sight. At best, symptomatic relief is given, control as in other chronic difficult-to-treat disorders like diabetes, hypertension, and IHD. Over time, likelihood of toxicity increases. Although our arsenal for RA treatment is powerful, long-term management and maintenance (control) remain major problems. Alternatives are needed. A summary of drug trials of Ayurvedic herbal formulations in the management of RA, is now presented.

### RA-1[18]

A standardized formulation, called RA-1, was prepared from purified plant extracts of *Withania somnifera, Boswellia serrata, Zingiber officinale*, and *Curcuma longa*, and evaluated over 16 weeks in a randomized double blind (RDB), placebo controlled, parallel efficacy, single center phase II drug trial (statistically designed with 80% power to detect significant difference at *P* < 0.05 and a dropout rate of 20%). Here 182 patients with active-on-chronic RA were enrolled and efficacy assessed as per the protocol. Oral paracetamol was permitted as a rescue analgesic. Patients were allowed a fixed stable dose of daily prednisolone not exceeding 7.5 mg. NSAIDs were not permitted and no diet or exercises were advocated. An intent-to-treat analysis failed to reach significance for primary efficacy response versus placebo, but did attain significance for: (i) increased proportion of patients with a 50% reduction in swollen joint count (95% CI,1.52, 29.90) and swollen joint score (95% CI,0.91, 28.73), (ii) a reduced RF titer (95% CI, –303.7, –2.72), and (iii) improved blood hemoglobin. And 39% in the RA-1 group versus 30% placebo showed the ACR (American College of Rheumatology) 20% improvement index[34] response (95% CI, –5.48, 24.59). Interestingly, RA-1 showed numerical superiority over placebo for every primary and secondary efficacy variable. Treatment groups reported only minor side-effects, 17 patients withdrew (active=9; placebo=8), none due to drug toxicity.

At weeks 32 and 54 of the continuing open label phase, patients showed significant improvement in all ACR core efficacy variables,[28] including a validated modified version of Stanford Health Assessment Questionnaire (HAQ) for Indian use.[18,35]

In conclusion, RA-1 was found to be a modest DMARD with an excellent safety profile. This trial, possibly for the first time, also demonstrated the feasibility of carrying out a state-of-the-art clinical validation of a herbal formulation derived from an ethnic medicinal system, with international collaboration.

### IRA-01[19]

A phase III drug trial: a 3-month, multicenter, RDB placebo-controlled phase, followed by a 9-month, single-center, open-label phase (with maximum enrolled patients). IRA-01 contained extracts of *Boswellia serrata* (Salai Guggul), *Trigonella foenum-graecum* (Fenugreek), *Linum usitatissimum* (Flaxseed), *Camellia sinensis* (Green tea), *Curcuma longa* (Turmeric), *Tribulus terrestris* (Gokshur), and *Piper nigrum* (Black pepper). Trial sample size was designed with 80% power (to detect 20% difference between active and placebo) and 5% Type I error (*P* < 0.05), dropout rate 20%. No NSAIDs, DMARDs, or prednisolone were permitted during the entire 1-year study duration. Paracetamol was allowed as a rescue analgesic on a need basis. Here 130 patients enrolled in the study.

During the RDB phase, IRA-01 showed greater improvement compared to placebo for all efficacy measures, but only achieved significance on physician global assessment of disease activity (Mann Whitney, Z=2.18; 95% CI of change –1.15, –0.01). RF titer fell significantly in the active IRA-01 group while it worsened in the placebo group. A strong placebo clinical response was evident (ACR 20 improvement for 60% patients on active drug, 53% on placebo). Thirty eight patients withdrew, with differences between active and placebo groups not reaching significance. Only minor side-effects were recorded during the entire study period, with no significant adverse alterations in routine hematology, biochemistry (renal and hepatic), or metabolic parameters attributable to IRA-01.

**Figure 1:**
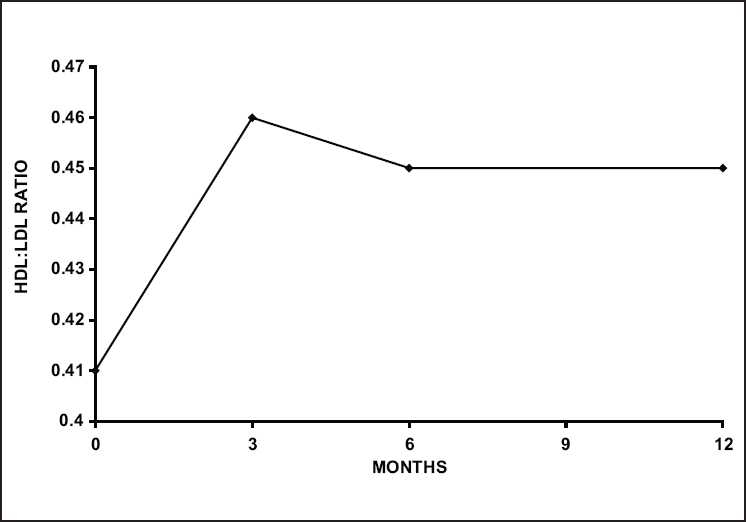
HDL/LDL ratio over time in patients completing 12 months of follow–up

After 3 months, 70 patients entered the open-label phase, of whom 58 (83%) completed 1-year follow-up. On completion, significant improvement in all efficacy variables including joint pain, swelling and Indian. HAQ was found (some *P* < 0.001). Here, 80% and 40% patients achieved ACR 20 and ACR 50 improvement response, respectively. Interesting incidental findings were significant changes in active arm serum HDL (increased) and LDL (decreased) levels during the RDB phase, which maintained throughout the year’s study [Figure 1]. Increases in serum protein with significant increase in serum albumin (95% CI –0.35,–2.90) were also observed at 12 months.

It was concluded that IRA-01 was a slow onset DMARD with modest efficacy, excellent safety profile, and improved quality of life.

### NMITLI/B1[20]

**Figure 2:**
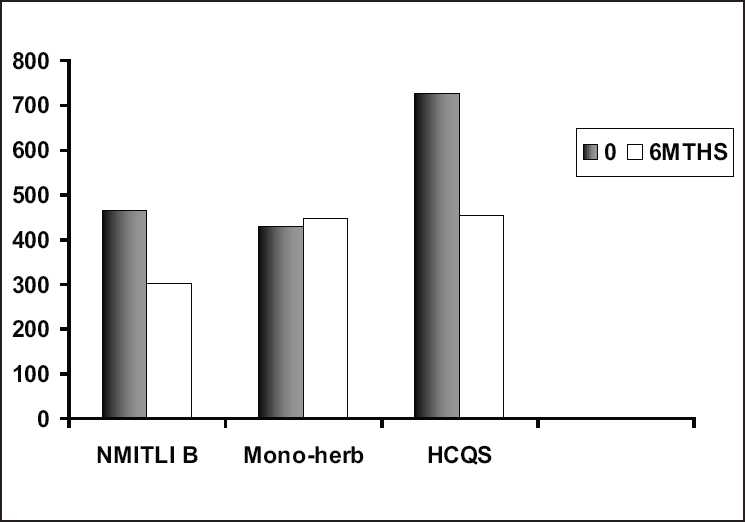
Rheumatoid Factor titer at baseline and completion in NMITLI/B clinical drug trial

B1, an Ayurvedic formulation developed under the NMITLI (New Millennium Indian Technology Leadership Initiative) project, contains plant extracts of Guduchi (*Tinospora cordifolia*), Ashwagandha (*Withania somnifera*), Gokshur (*Tribulus terrestris*), and Shunthi (*Zingiber officinale*). This trial compared B1 with a proprietary monoherb preparation (a formulation of Bhallataka, *Semecarpus anacardium*), and hydroxychloroquin (HCQS), a popular biomedical DMARD used to treat mild to moderate RA and long-term control. Total 121 patients with active RA were randomized into a three-arm (2 Ayurvedic and 1 HCQS), single-blind (investigator), parallel efficacy, 24-week, multicenter study. B-1 had earlier tested superior (not statistically significantly) to placebo for pain relief in another controlled trial of OA knees. Fixed oral doses of meloxicam (an NSAID, initial 12 weeks only), prednisolone (≤ 5 mg daily), and paracetamol rescue were permitted. An intent-to-treat analysis using ANOVA (significant *P* < 0.05) was carried out. All groups matched well at baseline. On completion, the groups did not differ significantly on any efficacy measure except physician global assessment. ACR 20% improvement response was demonstrated in 44%, 51%, and 36% of the B-1, HCQS and mono-herb, respectively. Pair-wise comparisons (corrected significant *P* < 0.02) found no differences between “B-1” and HCQS, but both HCQS and poly-herb “B-1” showed superior efficacy to mono-herb “BP.” Both B-1 and HCQS arms showed significant reductions in RF titer [Figure 2]. All groups reported mild adverse events, though gut and skin symptoms were higher for HCQS. And 34% patients withdrew; none due to AE, and none recorded a serious AE.

In probably the first ever head–head controlled RA comparison, this exploratory controlled drug study demonstrated similar efficacy to HCQS, but safety profiles like standardized Ayurvedic polyherbal formulations.

## OSTEOARTHRITIS KNEES[36]

OA is a common cause for chronic pains, disability, and poor quality of life and much more prevalent than RA in the community. Other than physiotherapy and exercises, the therapy options for OA in modern medicine are grossly limited to providing symptomatic relief using analgesics, including NSAIDs, or joint replacement in end-stage situations. Patients often self-medicate, consuming analgesics and NSAIDs for prolonged periods, running the risk of suffering life-threatening drug toxicity (esp. to gastrointestinal/renal/cardiovascular systems). Effective management needs drugs to repair and strengthen cartilage and prevent future damage. Glucosamine provides symptomatic pain relief, improved quality of life, and reduced cartilage damage.[37] Worldwide, it is used extensively to treat OA, but several other, well-designed studies have challenged its therapeutic role. A recent NIH trial demonstrated limited use of oral glucosamine in the treatment of OA.[38] Although long expected, there is a void of chondroprotective drugs. The ideal would provide pain relief and chondroprotection. In this light, an appraisal of drug trials of Ayurvedic herbal formulations in management of OA knees is presented:

### RA-11[21]

RA-11 was a further development of RA-1 (described earlier) showing improved potency, purity, and standardization, but with similar plant extracts. Interestingly, Ayurveda often uses similar formulations to treat diseases that are different under biomedicine’s classification, true to some extent of arthritis.

Total 90 patients of symptomatic primary OA knees with a post-analgesic wash-out pain on visual analog scale (VAS) more than 4 cm in either knee, or both, were enrolled in an RDB, placebo-controlled, parallel-efficacy, 32-week drug trial (80% power to detect 25% difference, type I error, *P* < 0.05, two sided). No concurrent analgesics, NSAIDS-oral/local, or intra-articular steroids were allowed. Patients received no dietary restrictions, or any specific physiotherapy program, but were encouraged to continue daily activities/exercise. Pain VAS (both knees) and WOMAC (Western Ontario McMaster Univ OA Index)[39] were “primary efficacy variables” (PEV). The WOMAC questionnaire evaluates knee function in daily life. The groups (active=45, placebo =45) were well-matched at baseline. The active group showed significant reductions (in a mixed model ANOVA) in weekly change rates for pain VAS (*P* = 0.006) and WOMAC [combined (*P* = 0.04), pain (*P* = 0.06), stiffness (*P* = 0.01), difficulty (*P* = 0.06)]. In an intention-to-treat analysis, active group mean PEV changes from baseline to weeks 16 and 32 showed significant improvement. Only mild AE were reported with no differences between groups. Routine laboratory monitoring (hematological and biochemistry) showed no important changes. And 24-hour urinary cortisol values (to detect any steroid compound/effect in RA 11) did not differ between groups at baseline or the week 16 endpoint (*P* = 0.7). Also, 23 patients (active 12, placebo 11) withdrew, none due to drug toxicity.

To conclude, RA11 showed significant efficacy compared to placebo over 32 weeks treatment of moderately severe symptomatic OA knees, demonstrating excellent safety profiles.

In the Open Label Phase 44 patients willing to continue beyond the randomized phase completed almost 2 years’ regular medication (unpublished data). Besides recording significant relief in pain and improved knee function, patients provided several other relevant observations – improved general health, energy level, sleep, and bowel habits. During follow-up, 25 initial active group patients (*n* = 45) had withdrawn by week 104. Causes were as follows: for 13, unsatisfactory therapeutic response; for 12 significant improvement; and none withdrew due to drug toxicity; only minor AEs were reported, mostly abdominal discomfort.

### NMITLI/C-01[22]

This NMITLI trial evaluated five standardized Ayurvedic formulations (A, B, C, D, and E), each containing *Shunthi (Zingiber officinale)-Guduchi (Tinospora cordifolia)* platform formulation along with selected plant extracts. Also 245 consenting eligible patients with symptomatic OA knees were randomized into seven arms (35 per arm) of an RDB, parallel efficacy, multicenter exploratory trial of 16-week duration. The trial was controlled for placebo and glucosamine sulfate use. No dietary or other restrictions were advised. Oral paracetamol was permitted for rescue analgesia and consumption monitored. Groups matched well at baseline. No between group differences emerged for patient withdrawals (43), or AEs (all mild). Intention-to-treat primary efficacy analysis found no significant differences for pain (weight bearing) or WOMAC (knee function). Placebo response was high. On pain relief criteria, Ayurvedic formulation “C” was selected for further development (*now labeled C-01*).

### NMITLI/C-02[23]

Ayurvedic formulation C-02’s composition, similar to C-01, improved its potency and standardization. A 6-week clinical dosing study, completed prior to the trial, demonstrated better clinical effectiveness with increased dose, i.e., further addition of *Guggul (Boswellia serrata)*; safety profile remained excellent (unpublished).

In a new study, two variations of C-02 (C-02/C-03) were selected and tested in a 24-week RDB, parallel efficacy, multicentre drug trial of equivalence, with oral Celecoxib and Glucosamine as comparators, statistically designed for sample size to achieve 80% power to detect differences at *P* < 0.05 significance. A priori, the range of equivalence for mean changes on PEV (active pain VAS, Indian version WOMAC [W/Likert version] pain and difficulty) was determined. Rescue analgesic (oral paracetamol) was discouraged. Post-consent, 440 eligible (active pain VAS> 4 cm) patients (median age/weight/active pain VAS = 55.5 years/65 kg/6.55 cm) with OA knees were enrolled and monitored according to protocol.

Groups matched well at baseline. Difference in mean change scores fell within target equivalence. Minor AE were reported in each arm (differences not significant); lesser AE were seen with C-02 formulation. Overall 28% patients withdrew with no between group differences. In conclusion, C-02 showed equivalent efficacy and better safety than glucosamine/celecoxib.

## DISCUSSION

Undoubtedly, modern medicine provides overwhelming symptomatic relief from pain and swelling for patients suffering from arthritis. However, idiosyncrasy and dose-related toxicity are major obstacles, especially where long-term management is required. Under these circumstances, the potential of Ayurvedic medicines should be tested, and, if substantiated, converted into real-life treatment paradigms constituting an effective Ayurveda-modern medicine interface. Ayurvedic drugs, as demonstrated by results of the controlled drug trials reviewed here, are capable of providing both short- and long-term relief for RA and OA patients.

Although efficacy at times is modest, safety is excellent. This should logically lead us to question “whether both modalities can be effectively used in conjoint treatment strategies?” providing effective solutions to difficult-to-treat chronic medical disorders. Implementing this requires all-out efforts from biomedical and Ayurvedic physicians to see eye-to-eye on human suffering.

Ayurvedic medicines are traditionally known to be safe; undoubtedly our Indian community craves to use them for relief. “Ayurveda is natural and safe” and “modern medicine is harmful” are deeply embedded perceptions. Modern medicine is overwhelmingly strong in emergency clinical situations. In Kerala, however, patients with any kind of emergency including poisonous snake bite are often first evaluated by Ayurvedic physicians who may even decide not to refer them any further (personal communication). The tremendous safety of Ayurvedic botanicals is very reassuring and forms the foundation of the much advocated “reverse pharmacology” approach,[40] where clinical validation proceeds in parallel to other experimental studies.

The ancient Ayurvedic sages viewed man not in isolation, but against the big picture of the universe. He was treated holistically. When establishing an Ayurveda–modern medicine interface, due care should be required not to subject Ayurveda to the reductionist approach, the foundation stone of modern medicine. Somehow, Ayurveda’s total treatment package seems to provide the body’s milieu interior with an opportunity to heal; no Ayurvedic formulation is an exception. The current report focuses only on Ayurvedic drugs.

The *Rasayana* branch of Ayurveda concerns “strengthening” the immune system, healing, and rejuvenation and, in the current context, is relevant to chronic arthritis. Above, we alluded to several unique facets of *Rasayana*, including experiments to garner supporting evidence. The Ayurvedic formulations used in the current trials contain several “*Rasayana*” plants. After long-term follow-up, especially OA-11 described above, several patients felt better and stronger with improved daily care functions, demonstrating the *potential of Ayurvedic formulations to improve quality of life*, often the central issue in chronic disease care. That we observed long-term clinical benefits extending beyond pain relief, and mere disease control, is therefore not surprising.

In a similar vein, in modern medicine, RA and OA knees are etiopathologically distinct, representing totally contrasting disorders. That the same formulations are efficacious for both, though intriguing, supports the *Rasayana* concept. For RA, *Rasayana* deals with immune modulation, of paramount importance in the biomedical scheme of treatment. All the RA clinical trials cited above found significant reductions in RF titter, a central biological event in RA pathogenesis. However, *Rasayana*s also promote anabolic effects of potential chondroprotective benefit to degenerating cartilage in OA. *Rasayana* may thus be a unifying hypothesis for RA and OA. The recently completed NMITLI project carefully evaluated and tested this and several other hypotheses.

What else can be surmised from the personal experience gained from the above Ayurvedic drug trials? The formulations proved better than placebo, though statistical significance was often not achieved. The selected formulations were rather ineffective at relieving pain in RA patients, though reduction in joint swelling was impressive (very low doses of prednisolone, used by less than a-third of subjects, were allowed). The IRA-01 trial also demonstrated that an Ayurvedic formulation and a biomedicine NSAID (meloxicam) can be given together without any drug interaction to treat RA. Patients in long-term follow-up studies required fewer analgesics, but such was not true for most OA patients, where trials allowed no rescue medication (paracetamol/NSAID).

One trial found an Ayurvedic formulation as good as HCQS, a popularly used DMARD for long-term control of mild-moderate RA. HCQS is well-known for skin and eye toxicity; our trial design was exploratory, sample size and duration being too limited to demonstrate such effects. Overall, trials demonstrated extensive anti-inflammatory and disease modification effects that future studies may augment. Perhaps most rewarding were our drug trials on OA knees: long-term clinical efficacy was comparable with biomedicine’s best, results suggesting that Ayurvedic formulations not only alleviate pain, but may heal and protect cartilage. Our NMITLI project colleagues have published experimental data[41] from *ex-vivo* chondrocyte cell culture demonstrating chondroprotective properties of *Phyllanthus emblica*.

It would be difficult, if not impossible, to provide irrefutable evidence validating Ayurveda’s entire system. Like several other groups, we believe that its medicines’ promise and potential is sufficiently validated by long-term clinical use over thousands of years. In a scientific context, however, critical questions of standardization and defining clinical indications need to be answered through modern experiments and clinical studies. New paradigms need defining and testing, so both systems can be used in parallel or tandem in given clinical situations. Bypassing early stages of conventional modern medicine models of drug development, the reverse pharmacology approach can directly test Ayurvedic medicines’ in controlled clinical situations, resulting in major savings in time and money.

## CONCLUSIONS

For several years, our group has engaged in validating anti-arthritic formulations using standard clinical drug trial protocols and GCP guidelines. Earlier trial designs were based on superiority. Often we were convinced of the clinical efficacy response, but failed to demonstrate statistical significance. Strong placebo responses have been a major dampener leading to several postulated reasons for unique placebo response in our Ayurvedic trials. Safety track records for Ayurvedic formulations have been excellent in them all. But something is amiss! We may speculate that the answer for proving efficacy may lie in identifying better methods to evaluate Ayurvedic drugs. Our recent NMITLI trials have begun to evaluate equivalence with modern medicine’s so-called gold standards, yielding superior safety profiles. Future studies should evaluate entire Ayurvedic care packages rather than medicines alone.

In summary, we described some basic understanding of Ayurveda, with special reference to arthritis. Biomedical and Ayurvedic approaches were described, identifying gray zones and common ground for combined management. Evidence for efficacy and safety of selected Ayurvedic formulations for standard clinical drug trials in arthritis was presented. The concept of “*Rasayana*” in Ayurvedic medicines for immune modulation and healing in difficult-to-treat disorders such as RA and OA was highlighted. We expect that we have provided enough thought and data to further establish an Ayurveda–biomedicine interface. This should eventually lead to a holistic evidence-based medical system encompassing the best of both worlds, and improving medical care. Ayurveda will thus address many needs unmet by modern medicine.[42,43]

## References

[1] Sharma PV (1994). Caraka Samhita (English translation).

[2] Valiathan MS (2003). The Legacy of Caraka.

[3] Srikanta Murthy KR (1993). Madhava Nidanam (roga viniscaya) of Madhavakara (English translation).

[4] Mishra B, Bhavmishra, Bhavprakasha Nighantu (1999).

[5] Frawley D, Lad V (1994). The Yoga of Herbs.

[6] Chopra A (2000). Ayurvedic medicine and arthritis. Rheum Dis Clin North Am.

[7] Chopra A, Patil J, Doiphode V, Patwardhan B (2001). Exploring ancient Ayurveda for rheumatology: Traditional therapy, Modern relevance and challenges. APLAR Bull.

[8] Sharma PV (1994). Caraka Samhita (English translation). Chikitsa Sthana, Chapter 28.

[9] Srikanta Murthy KR (1993). Madhava Nidanam (roga viniscaya) of Madhavakara (English translation), Chapter 22.

[10] Srikanta Murthy KR (1993). Madhava Nidanam (roga viniscaya) of Madhavakara (English translation), Chapter 28.

[11] Thatte U, Chhabria S, Karandikar SM, Dahanukar S (1987). Immuno-therapeutic modifications by Indian medicinal plants. Indian Drugs.

[12] Thatte UM, Dahanukar SA (1989). Immunotherapeutic modification of diverse infectious states by Indian medicinal plants. Phytother Res.

[13] Patwardhan B, Kalbag D, Patki PS, Nagsampagi BA (1991). Search of immunomodulatory agents: A review. Indian Drugs.

[14] Upadhyay SN, Upadhyay SN (1997). Therapeutic potential of immunomodulatory agents from plant products.

[15] Katiyar CK, Brindavanam, Tiwari P, Narayana DB, Upadhyay SN (1997). Immunomodular products from Ayurveda: current status and future perspectives. Immunomodulation.

[16] Rege NN, Thatte UM, Dahanukar SA (1999). Adaptogenic properties of six *Rasayana* herbs used in Ayurvedic medicine. Phytother Res.

[17] Budhiraja RD, Sudhir S (1987). Review of biological activity of withanolides. J Sci Ind Res.

[18] Begum VH, Sadique J (1988). Long term effect of herbal drug Withania Somnifera on adjuvant induced arthritis in rats. Indian J Exp Biol.

[19] Ghosal S, Lal J, Shrivastava R, Bhattacharya SK, Upadhyay SN, Jaiswal AK (1989). Immunomodulatory and CNS effects of sitoinosides IX and X, two new glycowithanolides from W somnifera. Phytother Res.

[20] Ziauddin M, Phansalkar N, Patki P, Diwanay S, Patwardhan B (1996). Studies on the immunomodulatory effects of Ashwagandha. J Ethnopharmacol.

[21] Sharma PV (1994). Caraka Samhita (English translation). Chikitsa Sthana, Chapter 28.

[22] Sharma PV (1994). Caraka Samhita (English translation). Chikitsa Sthana, Chapter 25.

[23] Srikanta Murthy KR (1993). Madhava Nidanam (roga viniscaya) of Madhavakara (English translation), Chapter 23.

[24] Chopra A, Lavin P, Patwardhan B, Chitre D (2000). Randomized double blind trial of an Ayurvedic plant derived formulation for treatment of rheumatoid arthritis. J Rheumatol.

[25] Chopra A, Saluja M, Patil J, Anuradha V, Bichile L (2004). IRA-01, An ayurvedic (Asian Indian) drug for rheumatoid arthritis (RA): Evaluation for efficacy and safety, and a probable lipid modifying effect. Ann Rheum Dis.

[26] Chopra A, Narsimulu G, Bichile L, Handa R, Raut A, Saluja M (2007). Comparing ayurvedic (Indian) herbal drugs and HCQS (hydroxychloroquin sulfate) in the treatment of rheumatoid arthritis (RA): A randomized, double blind, multi-centric exploratory drug trial of 24 weeks duration. Arthritis Rheum.

[27] Chopra A, Lavin P, Patwardhan B, Chitre D (2004). A 32-Week randomized, placebo-controlled clinical evaluation of RA-11, an Ayurvedic drug on osteoarthritis of the knees. J Clin Rheumatol.

[28] Chopra A, Raut A, Bichile L, G Narsimulu, Handa R, Saluja M (2006). A controlled drug trial to evaluate Ayurvedic derived Shunthi-Guduchi based standard formulations in the treatment of Osteoarthritis (OA) Knees: A Government of India NMITLI Arthritis Project. Ann Rheum Dis.

[29] Arvind Chopra, Saluja M, Venugopalan A, Naik P, Tillu G, Sarmukkaddam S (2008). A 24 week RDB multicentric trial to demonstrate equivalence between individual drugs for symptomatic treatment of OA Knees: Ayurvedic (Indian Asian), Glucosamine and Celecoxib. Ind Jour Rheum.

[30] Bellamy Nicholas (1993). Musculoskeletal Clinical Metrology.

[31] Alyce M, Oliver E, Clair WS, Klippel JH (2008). Rheumatoid Arthritis-Treatment and Assessment. Primer on the Rheumatic Diseases.

[32] Chopra A, Abdel-Nasser A (2008). Epidemiology of rheumatic musculoskeletal disorders in the developing world. Best Pract Res Clin Rheumatol.

[33] Joshi VL, Chopra A (2009). Is there an urban-rural divide?.Population surveys of rheumatic musculoskeletal disorders in the Pune region of India using the COPCORD Bhigwan model. J Rheumatol.

[34] Felson DT, Anderson JJ, Boers M, Bombardier C, Chernoff M, Fried B (1993). The American College of Rheumatology preliminary core set of disease activity measures for rheumatoid arthritis clinical trials: The committee on outcome measures in rheumatoid arthritis clinical trials. Arthritis Rheum.

[35] Chopra A, Gore A, Paranjape S, Edmonds J (1996). Modified Health Assessment Questionnaire: An Indian study for validity and relevance. Program of the 8th APLAR Congress, Melbourne, Australia, April 21-25, 1996.

[36] Sharma L, Klippel JH (2008). Osteoarthritis- Treatment. Primer on the Rheumatic Diseases.

[37] Reginster JY, Deroisy R, Rovati LC, Lee RL, Lejeune E, Bruyere O (2001). Long-term effects of glucosamine sulphate on osteoarthritis progression: A randomized placebo controlled Clinical trial. Lancet.

[38] Clegg DO, Reda DJ, Harris C CL, Klein MA, O’Dell JR, Hooper MM (2006). Glucosamine, chondroitin sulfate, and the two in combination for painful knee osteoarthritis. N Engl J Med.

[39] Chopra A (2004). Rheumatology: Made in India (Camps, COPCORD, HLA, Ayurveda, HAQ, WOMAC and Drug Trials). J Indian Rheum Assoc.

[40] Patwardhan B, Vaidya A, Chorghade M (2004). Ayurveda and natural products drug discovery. Curr Sci.

[41] Sumantran VN, Kulkarni A, Chandwaskar R, Harsulkar A, Patwardhan B, Chopra A (2008). Chondroprotective potential of fruit extracts of Phyllanthus Emblica in Osteoarthritis. Evid Based Complement Alternat Med.

[42] Engel LW, Straus SE (2002). Development of therapeutics: Opportunities within complementary and alternative medicine. Nat Rev Drug Discov.

[43] Mashelkar RA (2008). Second World Ayurveda Congress (Theme: Ayurveda for the Future)–Inaugural Address: Part III. Evid Based Complement Alternat Med.

